# Correction: Analysis of clinical trial registry entry histories using the novel R package cthist

**DOI:** 10.1371/journal.pone.0292761

**Published:** 2023-10-24

**Authors:** The PLOS ONE Staff

## Notice of republication

This article was republished on August 15, 2023, to correct code display problems caused by technical limitations and errors introduced at typesetting. These changes were not disclosed to the author or approved by the author prior to publication. Code boxes have been adjusted to support the formatting, and monospace styling has been applied to all code excerpts. Table 1 has been adjusted to fit all inline code excerpts on one line, and lighten cell shading for readability. A Unicode font has been applied to asterisks in the PDF. Unintended edits to code text that were introduced by the typesetter have been corrected. Exception: In the PDF, inline code excerpts may sometimes be auto-hyphenated, and line wrapping that occurs in the PDF’s inline code excerpts may sometimes translate to line breaks when copied/pasted. It is recommended that users copy/paste from the online article instead of the PDF.

The publisher apologizes for the errors. Please download this article again to view the correct version. The originally published, uncorrected article and the republished, corrected articles are provided here for reference.

## Supporting information

S1 File Originally published, uncorrected article. (PDF)

S2 File Republished, corrected article. (PDF)

## References

[1] Carlisle BG (2022) Analysis of clinical trial registry entry histories using the novel R package cthist. PLoS ONE 17(7): e0270909. 10.1371/journal.pone.0270909 35776915 PMC9249399

