# Supplementary material for: Analysis of clinical trial registry entry histories using the novel R package cthist
Source: PLoS One. 2022 Jul 1;17(7):e0270909. doi: 10.1371/journal.pone.0270909 (PMC9249399; doi:10.1371/journal.pone.0270909)
Supplement: Attachment — Submitted filename: Response to Reviewers.docx [file pone.0270909.s001.docx]

2022-04-11

Dear reviewers,

Thank you very much for your work and your thorough reviews of my paper. Your feedback was very helpful and feel that the package is a much better offering as a result of the changes that I was able to implement in response.

As a result of this review, I have published a new version of cthist (version 1.0.0) that includes these changes, which is now available on CRAN.

Please find below my responses to your comments in **bold** marked with “**>**”. All line numbers in this document refer to lines in the version of the manuscript that contains tracked changes between the revised version and the originally submitted one.

Best regards,

Benjamin Gregory Carlisle PhD

Berlin Institute of Health at Charité (BIH)
BIH QUEST Center for Responsible Research
Wiss. Mitarbeiter AG Translationale Bioethik
Anna-Louisa-Karsch-Str. 2, 10178 Berlin

benjamin.carlisle@bih-charite.de
Tel. +49 450 543 694
quest.bihealth.org

# Reviewer #1

This is an interesting paper that presents an R package that performs web scraping to get historical data on clinical trials from two registries : clinicaltrials.gov and DRKS. The code source of the package is open source on GitHub.
Some parts deserve a more elaborate description:
- the clinical trials API is not mentioned at all: from what I understand, the API does not provide the historical data and web crawling is the only way to get it, but still this should be clarified in the paper. The choice to use web crawling techniques has to be justified as using an API would be a much simpler way to proceed if it would be possible

- The mention of the API in the Introduction was admittedly brief, and has been expanded for clarity. See edits on lines 48-52.

- web scraping is a very useful technique but should be done in a "polite" way, to make sure not to disturb the underlying service / website. The way the package is used depends on the final user of course but this issue could be brought to the attention of the reader

- In response to your suggestion, I have re-implemented the functions that make calls to ClinicalTrials.gov or DRKS.de using the `polite` R package, to ensure that the sites' rules regarding scraping are observed, and to limit the number of requests. See commits https://github.com/bgcarlisle/cthist/commit/730c102a9d89f2787b00915494205298c4f433af and https://github.com/bgcarlisle/cthist/commit/cc30b1ba67f1406b7e4bbdcaaa176ea820c86ab5

- The 3 cases study are very interesting applications of the package. But only the third comes with results explaining the benefits of the historical analysis. At least some light results could be provided also for the first two cases study.

- I added a description of an already-published report in which these methods were used to inform the analysis of a cohort of Covid-19 trials after case study 1, and indicated why the methods were relevant. See lines 447-457.
- I have also added a description of an ongoing project that makes use of this package to after case study 2, and indicated why the methods were relevant. See lines 530-541.

- in Discussion / Future directions: clinicaltrials.gov and DRKS do provide access to webpages describing the historical changes : that may not be the case for other registries so this method may not be extended as such to any registry.

- I have qualified this part of the “Future directions” section to clarify that this package can only be extended to registries that also provide access to historical versions through their website, see lines 680-681.

# Reviewer #2:

First of all, I would like to thank the editor for the invitation to review this paper and draw my attention to this interesting R package. I think the package is a valuable addition to the R package ecosystem, as there are only very few, if any, packages that allow downloading clinical trial data (some older packages have been archived). Additionally, as the author has correctly pointed out, the usual methods for downloading trial data (e.g., the AACT) do not include the history of changes to a registry entry.

- Thank you! I'm glad that you find it useful.

The package itself could be described as rather minimalistic but worked well during my testing, and the paper describes the included functions quite well. The case studies illustrate briefly but convincingly how the package enables analyses that would otherwise require considerable manual work.

I could successfully run the code for all case studies and briefly tested the Shiny app, which also worked well. However, after reviewing the code and the article, I have some comments.

Comments regarding the package and code:

Comment: When I first tried the clinicaltrials_gov_dates function, it returned some correct dates but many NAs. It turned out that this was due to my language setting so that only some month names could be read in. I could avoid this error by temporarily changing the locale:
lct <- Sys.getlocale("LC_TIME") # backup
Sys.setlocale("LC_TIME", "C")
Maybe the function could do this automatically (and restore the original locale afterwards)? This will otherwise lead to frequent problems.

- I have addressed this issue in the manner you suggest. See the following commits: https://github.com/bgcarlisle/cthist/commit/2d910cad1c9452f148c3bec127e13e86219ebad9 and https://github.com/bgcarlisle/cthist/commit/452562ccb179467f08678515b94450c4f48c9eb7

Comment: The function should probably also warn if it returns Nas.

- I have added warnings in the case of returned NA's. See the following commit: https://github.com/bgcarlisle/cthist/commit/cc894ca7b8d4cc5093b265f15d9046366aa5467a

Comment: I realized the above when running the code line-by-line. I noticed that the line 'format("%Y-%m-%d")' in clinicaltrials_gov_dates seems to be superfluous, but maybe it is meant as a 'double-check' to make sure the dates are formatted correctly.

- This line converts the date into an ISO-8601 formatted character string, to prevent certain kinds of unexpected behaviour in writing R date objects to CSV output directly.

Comment: This comment is about both the article and the code. I think it is important to correctly name the returned value of clinicaltrials_gov_version (and the respective function for DRKS). The article often mentions 'lists' or 'data frames' (e.g., line 160), and the help for clinicaltrials_gov_version defines the returned value as a list, although it is actually a character vector.

Comment: In connection with the previous comment, I believe that it might indeed be better to let that function return a list instead of a character vector. First, lists are printed more nicely in the R console, and second, if the elements of the list were named appropriately (with 'outcomes', 'criteria', and so on), we could use $ to subset the returned list. Does returning the result as a character vector have any advantages?

- Thank you for this suggestion. I agree, and I have made this change as requested. The function now returns a named list, which is in accord with the package documentation and the manuscript. See the following commit: https://github.com/bgcarlisle/cthist/commit/ccb1b931ab365f9b9f5b15edec2f17c9cc03433d

Comment: I have tested the download function for ClinicalTrials.gov with 100 trials for a certain query and also the 93 trials on glioblastoma, as demonstrated in case study 1. However, all values for gender_based were missing. Is this plausible?

- The definition for the `gender_based` field can be found here: https://prsinfo.clinicaltrials.gov/definitions.html#EligibilityGender
- The `gender_based` field is meant to identify clinical trials where gender (not sex) is used as an eligibility criterion, and it appears to be left blank on the historical versions page in cases where inclusion is not based on gender (hence the apparently missing values). It would be very surprising for a glioblastoma trial to give gendered inclusion criteria, and so I am inclined to say that yes, the results you describe are plausible.
- That said, there was also a bug in the code that retrieved this data point that has now been fixed. See git commit https://github.com/bgcarlisle/cthist/commit/4bd4aac582cdf2f673fe5fc4598d57d9dedac393
- As an example, in the newest version of cthist, the following code returns “Yes” (NCT05306145 is a trial with gender based eligibility criteria):
- clinicaltrials_gov_version("NCT05306145", 1)$gender_based

Comment: It is good that the author has written unit tests for cthist. However, I think the tests are currently quite basic and could easily test the output more thoroughly. For example, test-clinicaltrials_gov_version.R could test for missing values and correct date format (or even some specific values), instead of only testing the length of the vector.

- This point is well-taken. I have implemented more extensive unit tests, one for each data point that is downloaded, see git commit: https://github.com/bgcarlisle/cthist/commit/e9db64c6e3600989f62d399664b155eca879c18a

Comment: It would be nice if ?cthist had a help page with a short overview of the package.

- This has been added. See the following commit: https://github.com/bgcarlisle/cthist/commit/be6a65a72f980a9fc566b6d410dc6f30cea489e2

Comment: ClinicalTrials.gov has announced an update to the website. Do you know if this will interfere with the scraper's functionality? Luckily, the beta version of the history page looks identical to the current version at first glance.

- The link on the beta version of the new version of ClinicalTrials.gov seems to redirect to the currently active history page, so they may not be planning to change that part at all, or if they are, they have given no clear indication (as of 2022-04-11). In any case, you are correct, this is one of the challenges in maintaining a web-scraper. I have mentioned this in the Limitations. The unit tests that you recommended and I implemented above will at least provide notice in case an update to the site breaks the web-scraping as it is currently implemented.

Comment: It seems a bit unusual to me that some of the functions, such as the *_dates functions) return "Error" and "Warning" as character vectors upon failure and print the warnings and errors using message(). I assume the intention is not to interrupt longer jobs if only very few tasks fail, but again, this is somewhat unusual because a character vector is the returned data format upon success. Typically, the warning() and stop() functions would be used here. Maybe the functions could have a parameter to let the user choose the behavior? I'm not sure, to be honest.

- Returning “Error” on failure is intentional. The output of this function is written to the CSV produced by the *_download() functions, to flag errors during download of large data sets without interrupting them, and to allow the function to remove bad or incomplete data after the fact.

Comment: The returned data class from the *_dates functions is a character vector, but the help states that it is a date vector (which would be better, in my opinion).

- This has been corrected. See the following commit: https://github.com/bgcarlisle/cthist/commit/51c33b4138355156df5a24c8ea077c25eef56b8c

Comment: Are you aware of any limits on automatic requests to ClinicalTrials.gov or DRKS.de? Some sites block web scrapers after a certain amount of requests. I don't think this is the case with these two sites, but it would be good to confirm that. Some users might download a very large number of trials.

- I have re-implemented the functions that make calls to ClinicalTrials.gov or DRKS.de using the `polite` R package, to ensure that the sites' rules regarding scraping are observed, and to limit the number of requests. See commits https://github.com/bgcarlisle/cthist/commit/730c102a9d89f2787b00915494205298c4f433af and https://github.com/bgcarlisle/cthist/commit/cc30b1ba67f1406b7e4bbdcaaa176ea820c86ab5

Comments regarding the article:

Comment: The case studies are short, but convincing. It would be good to show or mention at least some of the results for every case study instead of leaving this up to the reader.

- The results of the case studies have been expanded as you suggest. See lines 447-457 and 531-542.

Comment: Maybe it should be clearly mentioned that 'version 1' is the oldest version of a trial entry. It would be good also to say this on the R help pages.

- I have added this to the package documentation. See the following commit: https://github.com/bgcarlisle/cthist/commit/7e894a581fe5cd303aab8ef0d47a2273feda3b45

Comment: The package focuses on the history of registry entries, but I could imagine it being used for the general downloading of current entries. Since the rclinicaltrials package has been archived, there is no package with this functionality on CRAN, as far as I know. It would be helpful to briefly describe how to download only the most current entries. A 'trick' for doing so using slice_tail is included in one of the code examples but could be missed by readers easily.

- I can also foresee cthist being used in this way, however I would not recommend or encourage this as a use for this package. Downloading all versions of a clinical trial registry entry via web-scraping is extremely resource-intensive and this package does not download all data points for a clinical trial record. It would be better to encourage users to use the ClinicalTrials.gov API to download the current version.

Comment: Line 114 states "enrolment type (“Anticipated” or “Actual”), which are split into separate columns by the clinicaltrials_gov_download() function". It seems that actually 'Anticipated' or 'Actual' are included after the enrolment value in element 2 and additionally in element 5 of the returned vector. I assume element 2 should be only the numerical value?

- Element 2 of the returned vector (now a named list) contains a text string like “22 [Anticipated]”. This is the full text contained in the HTML element that is scraped from the page.

Comment: Is there a way to get the text 'cleaned' from line break code etc.? Could this be done by the package or are there other R packages that are better suited for that? Most users will probably want to clean these entries. A solution could be briefly mentioned.

- I believe conversion of the data from JSON to an R data frame will resolve the bulk of these issues. I have addressed this in the next comment.

Comment: I could successfully convert the JSON values that are returned by some of the functions. However, the article mentions in several places that, e.g., the outcome measures are 'data frames', although it seems to me R will convert the JSON to lists (when using rjson). Is this correct? I think returning an actual data.frame as a list element would actually be a more user-friendly solution than the current one, but I assume this was done to keep the output identical to the JSON entries in the CSV generated by the download function. Anyway, the article should be clear that the package does not return data frames or how the returned values could be converted to data frames.

- Using `fromJSON()` implemented by `jsonlite`, the data that is returned can be converted to a data frame. I have updated the text to clarify this. See lines 190-191.

Comment: Line 147: Maybe mention that 'Percentage change' refers to the percentage change of the length of the recruitment period.

- This has been added as suggested, see line 318.

Comment: Some references are missing DOIs. I also think that the citations of R packages should contain a link to CRAN, if applicable.

- These references have been updated as requested, see lines 726-781.

# Reviewer #3:

The author present a new package for the R statistical programming language, aimed as streamlining web scraping of two major clinical trial registries : ClinicalTrials.gov and DRKS.de. This package help retrieving change history of applications, a function which is not present in these registries API. It hints at an inclusion in reviewing of clinical trial, which would indeed be a useful complement to reported protocol changes.
In the discussion, the author press that the existence of this tool may reveal it's additional value, which is an important point.
This package is self-contained and usable without disturbance in multiple R paradigms. A version which do not request disk access and is able to operate fully on RAM may be prefered but is functional as is.

A) Regarding the article :

A.1) Abstract : I may advise to change "mass downloading" to "scraping", which is more precise while hinting at volume tolerance of the method. I am also surprised by the expression "the enterprise of human research", which I am not sure is widely used in english. As I am not a native speaker, this is just a question.

- I have changed “mass downloading” to “automated scraping.” As for “the enterprise of human research,” referring to the “scientific enterprise” or the “research enterprise” is reasonably common within a certain part of the research ethics literature, e.g. https://www.ncbi.nlm.nih.gov/pmc/articles/PMC4516403/

A.2) In the introduction, the author write "Certain research questions [...] are not possible or not feasible to address without a means of accessing and analyzing the history of changes for an entire cohort of clinical trials in a systematic way." I would be pleased to be presented with one or two examples.

- I have added an example to clarify. See lines 43-46.

A.3) Again in the introduction, the author write "There is no API access to historical clinical trial registry data for
ClinicalTrials.gov or DRKS.de." As there is in fact an API for both of these registries, I would have like a more precise description of the limitation of theses API and the need of this webscraping package.;;
When visiting ClinicalTrials.gov API webpage, it call up the Clinical Trials Transformation Initiative (CTTI)'s Database for Aggregate Analysis of ClinicalTrials.gov (AACT). Maybe add a sentence to discuss the objective of this base and the limitations motivating the creation of the cthist package ?

- You are correct that there is indeed an API to access the *current* version of ClinicalTrials.gov registry records, however there is no API access to *historical* clinical trial registry data for ClinicalTrials.gov. Older versions of clinical trial registry entry versions are not accessible for downloading using the API, which is the purpose of this R package. This point has been clarified in lines 48-52.
- I am unaware of any API for DRKS. Would you be able to supply a link to documentation?

A.4) In the method part, the details concerning the *_version functions seems shared between both, and the paragraphs have many repetition making reading it slightly laborious. Does the integration of more technical parts in the table 1 make in fact for a more or less concise and reader-friendly presentation ?
Complementary, regexes are mostly simple, but as a "write only" language it may be best to systematically add a translation in plain english. As there is mostly 3 different regexes, they may be substituted in the text by the plain english translation.

- This section has been heavily edited for brevity and clarity, and the repetitive parts have been moved to Table 1 as much as possible. See lines 130-157, 159-164, 213-249 and Table 1.

A.5) Again in the method part, I would like to have more precision on the two following affirmations :
"These functions also implement automated error-checking on completion, in order to ensure the accurate retrieval of large sets of clinical trial registry entry histories. If any version of the clinical trials to be downloaded returned an error, these functions return FALSE, otherwise they return TRUE." and "These functions also implement automated checking for already-downloaded data when starting, to allow for re-starting partially completed downloads."
Which further details on what type errors are checked and to what extend : do the function only return the website error ? Are errors sources furthermore explored ? etc.
For the second and from what I read in the code, it only check if a data is already present and complete, accelerating complementary download more than explicitly "allowing" it ?

- This has been expanded for clarity. See lines 280-296.

A.6) For Case study 3, even as it is just an example, why not keep trials which and describing status completion as completed, terminated or censored at extraction date (and statut regarding the exceeding of forecasting end date) ?

- As mentioned in the manuscript, “A trial that was registered yesterday, for example, may yet go on to be withdrawn, if it were given the same follow-up time as the trials in the sample that were registered five years ago. By failing to account for variable follow-up, our hypothetical researcher is systematically introducing bias into their sample and may produce a misleading count of the number of stopped trials.”
- The point of this analysis is to show that results may change if data is properly truncated to only include data points where there is adequate follow-up time, and that such an analysis would be difficult without cthist.

A.7) In the discussion part : As this package is also aimed at researchers not accustomed with web scraping, I would advise to add one or two paragraphs introducing to the basics of web scraping : differences between using an API and scraping a page, the risk of brutal changes and end of service, websites and other users respect regarding requests volume and frequency, risk of IP ban and other countermeasures, etc.

- I have expanded the Limitations to address this. See lines 661-673.

B) Regarding the code examples :

B.1) For all code examples, adding a (succinct) comment on each line code may be useful for readers not accustomed with the pipe workflow and tidyverse-specific function.

- I have expanded the comments in order to make the workflow more explicit for those unfamiliar with `tidyverse`. See lines 336-337, 360-366, 395-400.

B.2) This phrase is really hard to read and understand until later in the code, and may benefit of a refactoring centering on objectives in the style of "extract initial definitions of start and completion date"
"## Define the start date at launch and completion date at launch to be
\## the start date and completion date reported on the first version of
\## the trial where that version's overall status is listed as
\## "Recruiting" "

- This comment has been edited for clarity.

B.3) In the pipe `readr::read_csv("SearchResults.csv") %>% select(`NCT Number`) %>% pull() `, using `pull()` without argument is at risk. `readr::read_csv("SearchResults.csv") %>% pull(`NCT Number`)` should be more reliable.

- This has been changed as per your suggestion, see lines 339, 490 and 589.

B.4) The definition of "1 year" beeing variable in number of days and `followup <- 365` carrying a risk of error, it may be beneficially replaced by `dmy("21-01-2021") + lubridate::years(1)`, taking care of leap year.

- I have made this change as per your suggestion. See lines 325, 349, 578 and 599.

B.5) In code box 2, the use of an external rle object may not be apparent for readers. The author may add an reason for the use of run length, or maybe go for a whole-in-one approch as in :
```
hv %>%
mutate(outcome_run =
paste(drksid, primary_outcomes, secondary_outcomes) %>%
{. != lag(.)} %>% coalesce(TRUE) %>% cumsum()
) %>%
group_by(outcome_run) %>%
slice_head() %>%
ungroup()
```

- Thank you for this suggestion. I have expanded the code comments to clarify the use of the `outcome_runs` rle object to make its use more apparent for readers, but I have not changed it to the all-in-one approach that you outlined, because it seems less readable to me. See lines 500-501 and 508-509.

B.6) In the code box 3, it is possible to additionally reduce redundancy by using `stopped = overall_status %in% c("Suspended", "Terminated", "Withdrawn")` in line 491.

- Thank you for this suggestion. I have rewritten this part of the code in the manner that you suggest. See lines 621-626.

C) Regarding the package in itself :
As a first point, I wish to emphasize the global quality of the package design and the use of dependency for import of other packages. The following comments may in no point influence the publication decision as they relate to the package in itself and not the article presenting it. The three major limitation that I see as of now are :

C.1) The lack of defensive programming in the current state, with no testing of function argument and thus no meaningful error code and no catch of error before handling to the registry website. As NCT numbers format is strictly defined, adding verification that a character vector of length one and corresponding to a defined regex may be a critical add, ensuring that calls like `clinicaltrials_gov_version("bad_id", "2,8")` or `drks_de_version("DRKS0000000000005219", glm(Species ~ Sepal.Length, data = iris))` have informative errors.

- Thank you for this suggestion. I have implemented it as requested. All functions now check for well-formed input and provide informative error messages if this is not the case. See the following commits: https://github.com/bgcarlisle/cthist/commit/cea0daf035e18ba96d452d7feb4f77a8b540086c and https://github.com/bgcarlisle/cthist/commit/9e5b1ba42d8106c3fd992595796cb213e187a8ff

C.2) For all extracted dates, filling in missing data may be misleading in creating false precision. I would advise to either : issue a warning or, preferably, keep dates as plain text and let the user deal with imprecision in day number in the way that is more effective in the specific study context (first day, last day, interval, discard, etc.)

- For the dates on ClinicalTrials.gov that are sometimes reported as month-only, I have added new columns that indicate the precision with which the dates are originally reported, to address this concern. See the following commit: https://github.com/bgcarlisle/cthist/commit/72cc25034118d487f703fbe8ff59c6d928fbd89d

C.3) By the actual design, the *_download() functions request a writing disk access, which : 1) may be difficult in some secure analysis environnement, and 2) is a deviation on the general R objective of "no side effect". For the cleanest effect, it may be preferable to rewrite the *_download() to only batch call the *_version() functions and let the user choose how to keep its data and control it's storage. A simple alternative may be to change `output_filename` default value to `output_filename = tempfile()`, storing values in RAM, and make the function return the data frame (invisibly if needed) in place of an indication of success or failure.

- I have made the changes you suggest, see the following commit: https://github.com/bgcarlisle/cthist/commit/bff86eff83c1685e25144fe134a5e69195760121

Additional points that may streamline the package starting and usage :

C.4) When screening the code, the *_version() and *_dates() function seems to be backend for by *_download() function. If they are not intended to have separated use, they may be keeped as internal function and only *_download() function may be exported : https://r-pkgs.org/namespace.html . This way there will also be less function help-pages to write.
In addition, *_version() function are the ones actually downloading the informations and return it in R and *_download() function map *_version() functions and write it to the disc, which is slightly misleading.

- While these functions *are* primarily intended to work in conjunction with the *_download() functions, they may also be useful by themselves, and so I am inclined to export them.

C.5) If *_version() function are keeped exposed, adding a default version number value may be useful, for example `versionno = 0`, and defensive programming added to it too.

- As per your suggestion, default version numbers and defensive programming have been added to these functions. See the following commits: https://github.com/bgcarlisle/cthist/commit/955c04a401b9fff6925aa2e98dbeb80830e14fe7 and https://github.com/bgcarlisle/cthist/commit/9e5b1ba42d8106c3fd992595796cb213e187a8ff and https://github.com/bgcarlisle/cthist/commit/cea0daf035e18ba96d452d7feb4f77a8b540086c

C.6) The help files may be slightly thin, and adding one of the article example may be of great benefit, especially the explanation of how to get a csv of search result and not having to manually copy the individual NCT numbers. If it is simple enough, do the author plan to add a function taking a research string as an argument and returning the NCT numbers ?

- I have expanded the help to include instructions for how to retrieve the TRNs without manually copying them as per your suggestion. See the following commit: https://github.com/bgcarlisle/cthist/commit/b4718e534d2a330a8a2c57d57e0e59dc19fe5bcb
- There are many other methods for searching ClinicalTrials.gov and DRKS including their web sites/API. I'm afraid that it is outside the scope of this project to duplicate these tools.

C.7) To avoid IP ban or accidental DOS attack, is there a timer between two requests by *_version() functions ? Adding one, setting it as arbitrarily large (for example 1 second) and presenting it as an argument would be useful at the same time as warning against error and as teaching.

- As per your suggestion, I have re-implemented the functions that make calls to ClinicalTrials.gov or DRKS.de using the `polite` R package, to ensure that the sites' rules regarding scraping are observed, and to limit the number of requests. See commits https://github.com/bgcarlisle/cthist/commit/730c102a9d89f2787b00915494205298c4f433af and https://github.com/bgcarlisle/cthist/commit/cc30b1ba67f1406b7e4bbdcaaa176ea820c86ab5

# Reviewer #4:

Many thanks for the opportunity to review this piece explaining a new R package to examine historic trial information at ClinicalTrials.gov and the DRKS.

The purpose of this package is well described and justified. Working with historic registry data is something I have had to do, and write ad hoc code to handle (though in Python for my cases), for a number of my projects. Creating a package that handles at least some of these aspects of this type of task is certainly welcome and valuable. I have separated my comments into three parts covering my use of the package, the code, and the text of the manuscript. I note some minor points all around.

Comments on Usage:

While R is not my primary language I was able to install the package from CRAN and work through the various functions. For the author’s reference, I was working in R v4.0.5 within RStudio v1.4.1106 on a Mac running 10.15.17.

I cross checked outputs with a few sample trials from each registry and found no discrepancies. I admit, my lack of experience in R likely limited my ability to play around with the data in more depth, but the outputs are all very simple. It’s either a vector of dates, or a character vector containing strings of the variables in the relevant fields that can be easily outputted to CSV. The more complex fields are organized and tagged logically (in a way similar to a Dictionary in Python).

One note is that when I ran the example function in the documentation for downloading multiple ClinicalTrials.gov records: `clinicaltrials_gov_download(c("NCT02110043", "NCT03281616"), "test_review.csv")` it worked just fine to actually download the data and output the file but I got a parsing error on each row in which it expected 18 columns and got 19. The output is copied below. This might be unavoidable for some technical reason, but if that is the case perhaps note this in the documentation so that people aren’t thrown off by the errors during use. The DRKS example did not do this.

clinicaltrials_gov_download(c("NCT02110043", "NCT03281616"), "test_review.csv")

022-03-09 12:49:00 NCT02110043 processed (8 versions, 50%)
2022-03-09 12:49:06 NCT03281616 processed (2 versions, 100%)
Warning: 10 parsing failures.
row col expected actual file
1 -- 18 columns 19 columns 'test_review.csv'
2 -- 18 columns 19 columns 'test_review.csv'
3 -- 18 columns 19 columns 'test_review.csv'
4 -- 18 columns 19 columns 'test_review.csv'
5 -- 18 columns 19 columns 'test_review.csv'
... ... .......... .......... .................
See problems(...) for more details.

[1] TRUE
Warning messages:
1: Unnamed `col_types` should have the same length as `col_names`. Using smaller of the two.
2: Unnamed `col_types` should have the same length as `col_names`. Using smaller of the two.

Otherwise, I think this is all relatively straightforward and anyone with familiarity working in R should be able to get from these simple functions to workable data relatively easily.

- Thanks for pointing this out! There appears to have been a mismatch in the number of columns that was written to disk and the number of columns that I told the script to look for when checking after download, which caused these warnings. This has been corrected; see the following commit: https://github.com/bgcarlisle/cthist/commit/4eabe468b82e55dbaa71ebca2746a7a3d80b2a79 (The number of columns has increased since the previous version, because there are now “date precision” columns to address another issue, above.)

Comments on the Code:

Once again, slightly limited by my knowledge of R here, but I did take a look over the code for the package on GitHub. Since, as the author notes, there is no API for either registry that can be used for historic data, this package essentially runs a scraper in the background to gather the relevant data from the history pages. This is noted in the limitations of the paper, but you might also want to include in your documentation so people can understand that very large queries might cause issues at the host registry.

- To mitigate this concern, I have re-implemented the functions that make calls to ClinicalTrials.gov or DRKS.de using the `polite` R package, to ensure that the sites' rules regarding scraping are observed, and to limit the number of requests. See commits https://github.com/bgcarlisle/cthist/commit/730c102a9d89f2787b00915494205298c4f433af and https://github.com/bgcarlisle/cthist/commit/cc30b1ba67f1406b7e4bbdcaaa176ea820c86ab5

While I’m not in a position to comment on any by-line bugs or enhancements, given my own experience scraping historic registry data, I was able to follow how each function broadly worked, including how the `download` functions call the other functions to operate and it all made sense from an implementation perspective. I especially like the feature to re-check for partial downloads to not waste time and pings of the registry if a scrape is interrupted.

I was able to get all the code examples to run and produce expected results on my own.

- This is very encouraging, thank you for your close attention!

Comments on the Text:

Since you have a table that matches each extracted field to it’s CSS selector, I think you can simply point to that rather than also write it out in words from lines 108-163 and 175-206. If you want to keep details of any context you feel is necessary about the extractions in prose, that is fine but there’s no need to keep repeatedly referencing the CSS selectors and HTML elements used when the Table does that much more efficiently for anyone who is interested. Otherwise it really hinders the readability of that section.

- This section has been re-written for brevity and clarity as per your suggestion. The repetitive sections have been removed and Table 1 has been expanded to include those details. See lines 130-157, 159-164, 213-249 and Table 1.
