# Supplementary material for: Analysis of clinical trial registry entry histories using the novel R package cthist
Source: PLoS One. 2022 Jul 1;17(7):e0270909. doi: 10.1371/journal.pone.0270909 (PMC9249399; doi:10.1371/journal.pone.0270909)
Supplement: Attachment — Submitted filename: 2022-06-17-rebuttal.docx [file pone.0270909.s002.docx]

2022-04-11

Dear reviewers,

Thank you very much for your work and your thorough reviews of my paper. Your feedback was very helpful and feel that the package is a much better offering as a result of the changes that I was able to implement in response.

I have responded to the remaining minor changes requested below.

Please find below my responses to your comments in **bold** marked with “**>**”. All line numbers in this document refer to lines in the version of the manuscript that contains tracked changes between the revised version and the originally submitted one.

Best regards,

Benjamin Gregory Carlisle PhD

Berlin Institute of Health at Charité (BIH)
BIH QUEST Center for Responsible Research
Wiss. Mitarbeiter AG Translationale Bioethik
Anna-Louisa-Karsch-Str. 2, 10178 Berlin

benjamin.carlisle@bih-charite.de
Tel. +49 450 543 694
quest.bihealth.org

# Reviewer #1

(No Response)

# Reviewer #2:

The author has made extensive changes to both the paper and the package. Both are in a much-improved state compared to the previous submission. My comments have been addressed sufficiently, and I would like to thank the author for his detailed responses. In my opinion, the article is now acceptable for publication.

- Thank you!

# Reviewer #3:

I would like to thanks the author for his responses and clarifications. The context and the workflow benefit from more in-depth description and the integration of the "polite" package is a great quality improvement of the cthist package.
I now only have two tiny comments :
- In response for the inquiry about DRKS API : I had found this aggregator service via clarivate https://www.cortellislabs.com/page/?api=api-CLI . It may not be in fact sufficient for the intended use, but may be worth mentioning.

- A reference to the 3rd-party API has been added to the manuscript. See lines 51-54.

- The code comment referred in B.2) "## Define the start date at launch [...]" is mentioned as edited but have no tracked change. Have one of the intended change been slipped away between versions ?

- A comment beginning “## Define the start date at launch” appears twice in the manuscript. In both cases, a clarifying paragraph has been added afterward. See lines 354-369 and 390-402.

Thanks again for this new tool in the R environment and the opportunity to review this package

- Thank you for your review!

# Reviewer #4:

(No Response)
